# Identification and development of a functional marker from *6*-*SFT*-*A2* associated with grain weight in wheat

**DOI:** 10.1007/s11032-015-0266-9

**Published:** 2015-01-30

**Authors:** Aiqin Yue, Ang Li, Xinguo Mao, Xiaoping Chang, Runzhi Li, Ruilian Jing

**Affiliations:** 1 The National Key Facility for Crop Gene Resources and Genetic Improvement/Institute of Crop Science, Chinese Academy of Agricultural Sciences, Beijing, 100081 China; 2 Agronomy College, Shanxi Agricultural University, Taigu, 030801 China

**Keywords:** Haplotype, Marker development, Association analysis, Thousand-grain weight, *6*-*SFT*, *Triticum aestivum*

## Abstract

As a class of water-soluble, fructose-based oligo- and polysaccharides, fructans are major nonstructural carbohydrates and an important carbon source for grain filling in wheat (*Triticum aestivum* L.). Four enzymes are involved in fructan synthesis in higher plants, and 6-SFT is a key enzyme in fructan biosynthesis. In this study, thirteen single nucleotide polymorphisms were detected in *6*-*SFT*-*A2* in 24 wheat accessions, forming three haplotypes. Two cleaved amplified polymorphic sequence markers developed based on polymorphisms at sites 1870(A-G) and 1951(A-G) distinguished the three haplotypes. *6*-*SFT*-*A2* was located on chromosome 4A, between markers *P2454.3* and *P3465.1* in a doubled haploid (DH) population derived from the cross Hanxuan 10 × Lumai 14. The DH population comprising 150 lines and a historical population consisting of 154 accessions were used in a *6*-*SFT*-*A2* marker–trait association analysis. The three haplotypes were significantly associated with thousand-grain weight (TGW) under rainfed conditions. *Hap*III had a significant positive effect on TGW. There were significant differences between the Hanxuan 10 and Lumai 14 genotypes in both rainfed and irrigated environments. The average TGW of Lumai 14 (*Hap*III) was higher than that of Hanxuan 10 (*Hap*I). The frequencies of *6*-*SFT*-*A2* *Hap*III in cultivars released at different periods showed that it had been strongly positively selected in breeding programs. The preferred *Hap*III for TGW occurred at higher frequencies in Gansu, Beijing, Shanxi, and Hebei than other regions in northern China.

**Electronic supplementary material:**

The online version of this article (doi:10.1007/s11032-015-0266-9) contains supplementary material, which is available to authorized users.

## Introduction

Wheat (*Triticum aestivum* L.) is one of the most important staple food crops globally. With an increasing world population, it is estimated that the global demand for wheat will increase by a further 40 % before 2020 (Rajaram 2005). Therefore, higher yield is a predominating objective in wheat breeding programs. Water-soluble carbohydrates (WSC) accumulated in wheat stems constitute an important carbon source for grain filling. Variation in stem WSC concentration among wheat genotypes is one of the genetic factors considered to influence grain weight and yield in water-limited environments (Asseng and van Herwaarden 2003; Ruuska et al. 2006; Xue et al. 2008). Therefore, high WSC concentration is considered to be a potentially useful trait for improving wheat grain weight and yield in water-limited production environments (Blum 1998; Asseng and van Herwaarden 2003; Ruuska et al. 2006; Foulkes et al. 2007; McIntyre et al. 2011, 2012).

Fructan is the dominant form of WSC in wheat stems (Ruuska et al. 2006). At the stage of maximum WSC content, fructans represent 85 % of the WSC in wheat stem internodes (Blacklow et al. 1984). Genotypic differences in stem WSC concentration at anthesis are attributed mainly to the fructan component (Ruuska et al. 2006; Xue et al. 2008). Fructan accumulation in wheat stems continues during stem growth and anthesis and then declines significantly while contributing to grain filling (Pollock and Cairns 1991; Schnyder 1993; Goggin and Setter 2004). Fructan has also been assigned a possible role in conferring tolerance to terminal drought of wheat (Wardlaw and Willenbrink 2000; Foulkes et al. 2007; Volaire and Lelièvre 1997).

Fructans are a class of water-soluble, fructose-based oligo- and polysaccharides. Four enzymes, sucrose–fructan 6-fructosyltransferase (6-SFT), sucrose–sucrose 1-fructosyltransferase (1-SST), fructan–fructan 1-fructosyltransferase (1-FFT), and fructan–fructan 6G-fructosyltransferase (6G-FFT), are involved in fructan synthesis in higher plants (Vijn and Smeekens 1999). 6-SFT either transfers fructosyl residues to a fructan (i.e., to 1-kestose) in (2–6) linkages or produces 6-kestose (if only sucrose is available as a substrate). Fructans in wheat are mainly the graminan type, that is predominant b-2, 6-linked fructosyl-units with shorter b-2, 1-linked branches (Bancal and Triboï 1993; Ritsema and Smeekens 2003; Chalmers et al. 2005). 2–6 fructosyl-fructose linkages are by far the most prevalent in wheat fructans. Therefore, most of the carbon flux from sucrose to fructan in wheat is mediated by 6-SFT (Duchateau et al. 1995; Vijn and Smeekens 1999). Xue et al. (2008) showed that stem fructan concentrations at anthesis had a strong positive correlation to mRNA levels of *6*-*SFT* in wheat stems during stem growth and anthesis.

With recent advances in sequencing and genotyping technologies, the knowledge of genetic and genomic variation has rapidly increased (Berard et al. 2009). A nucleotide base is the smallest unit of inheritance; hence, single nucleotide polymorphisms (SNPs, which include single base changes and small insertions/deletions) provide the ultimate level of molecular genetic marker variation. Furthermore, SNPs are valuable molecular genetic markers due to both their abundance and their relative stability in the genome and can be applied as perfect molecular markers when identified within genes underlying observed traits (Edwards et al. 2007; Lv et al. 2013; Uribe et al. 2013). They provide valuable markers for the study of agronomic or adaptive traits in plant species using strategies based on genetic mapping or association mapping (Edwards et al. 2007). Association analysis has been applied in a number of species (Gupta et al. 2005). In wheat, association analyses have been employed to associate individual candidate gene polymorphisms with phenotypic variation in grain weight and photoperiod (Su et al. 2011; Guo et al. 2009). Thus, by association analysis, causative molecular polymorphisms can be identified and functional markers can be derived from them (Andersen and Lübberstedt 2003).

The genomic sequence of *6*-*SFT* was isolated (GenBank No. FJ228688.1) by Gao et al. (2010) from Triticeae plants. We detected polymorphisms in the *6*-*SFT*-*A1* locus, mapped it on chromosome 4A, and revealed that SNP in *6*-*SFT*-*A1* gene was associated with wheat seedling drought resistance (Yue et al. 2011). The objectives of this study were to (1) identify *6*-*SFT*-*A2* gene sequence polymorphisms among genotypes; (2) develop a functional markers for *6*-*SFT*-*A2* and map it on a chromosome; (3) reveal relationship between the marker and important agronomic traits by association analysis using a doubled haploid population and a historical population; and (4) identify superior *6*-*SFT*-*A2* haplotypes for marker-assisted selection in wheat breeding program.

## Materials and methods

### Plant materials

Three diploid progenitor species of common wheat, *T. urartu* (AA) accession UR 203, *Ae. speltoides* (SS, closely related to the B genome) Y2041 and *Ae. tauschii* (DD) Y215, and *T. durum* (AABB) DS 6 were used for identifying genomic origins. A set of nulli-tetrasomic lines developed in Chinese Spring was used for chromosomal location of the target gene.

The 150 doubled haploid lines (DHLs) derived from cross Hanxuan 10 × Lumai 14 were used for genetic mapping and gene–trait association analyses. Both parents Hanxuan 10 and Lumai 14 are Chinese wheat cultivars (Jing et al. 1999).

A historical population consisted of 295 wheat accessions (Supplementary Table S1) released during different decades was used for investigating the distribution of target gene haplotypes, and 154 of them were used for functional validation of the *6*-*SFT*-*A2* markers by association analysis. Of the total accession set, 272 were from China, 1 from Romania, 2 from Italy, 3 from Australia, and 15 from CIMMYT, Mexico. All accessions were kindly provided by the China National Genebank, Beijing.

### Field trials and measurement of grain traits

The DHLs were planted in the wheat growing seasons of 2001, 2005, 2006, 2009, 2010, and 2011, and the historical population was planted in 2008 and 2009 at Changping, Beijing (116°13′E; 40°13′N), the experiment station of the Institute of Crop Science, Chinese Academy of Agricultural Sciences. The experimental field was managed under drought stress (DS) and well-watered (WW) conditions. DS treatment was represented by rainfed conditions. The rainfalls from the beginning of October (at seeding) to mid-June (harvest) were 181, 100, 124, 155, 188, 131, and 180 mm for each growing season in the order of years. The WW treatment was irrigated with 750 m^3^ ha^−1^ at four growth stages: pre-overwintering, jointing, flowering, and grain filling, if there was not sufficient rainfall in each corresponding period. All accessions were planted in 2- or 4-m four-row plots with 30 cm spacings. Field management followed local practices. One thousand-grain weights (TGWs) were recorded after harvest.

### DNA extraction, primer design, PCR, and sequencing

Genomic DNA was extracted from young leaves of 10-day-old seedlings using the phenol/chloroform method (Sharp et al. 1988).

Based on the known sequence (GenBank No. FJ228688.1; Gao et al. 2010), the primer pair F1/R1 (F1: 5′-TACCAAACTCTCTTAGAGTTCACGAGGG-3′, R1: 5′-CACGAGTCCACTC TCCCAAACAACAATA-3′) was designed to amplify the *6*-*SFT* gene. Based on DNA variations among the genomic sequences of the *6*-*SFT* gene in each genome, one pair of A genome-specific primers F2/R2 (F2: 5′-CTCTCTAGACATAATCAAAAGGGA-3′, R2: 5′-TTCTTTGATC CAATGTAGCTTCA-3′) was designed. Primers were designed by the software Primer Premier, version 5.0 (Premier Biosoft International, Palo Alto, CA), and all primers were synthesized by Beijing Augct Biological Technology Co., Ltd (http://www.augct.com).

PCR were performed in total volumes of 15 μL, including 3 pmol of each primer, 100 μmol of each dNTP, 30 ng genomic DNA, and 1.5 unit *TransStart*™ *FastPfu* DNA Polymerase (TransGen Biotech Co. Ltd). The PCR procedure was initial denaturation at 95 °C for 5 min, followed by 35 cycles of 95 °C for 1 min, annealing (55 °C) for 45 s, and extension at 72 °C for 3 min; with a final extension of 72 °C for 10 min. The PCR products were separated by electrophoresis in agarose gels, and the target bands were recovered and cloned into the pEASY-Blunt simple vector (TransGen Biotech Co. Ltd). Twelve positive clones of each accession were sequenced.

DNA sequencing was performed on a 3730XL DNA Analyzer (ABI) with the following program: initial denaturation at 96 °C for 1 min, 30 cycles of 96 °C for 10 s, 50 °C for 5 s and 60 °C for 4 min. To screen the whole sequence of *6*-*SFT*-*A2*, both M13 and two pairs of overlapping primers (GH-1: 5′-GCAAAACAGGGGAAAACAG-3′, GH-2: 5′-GGTACCACATGTTCTTC CAG-3′, GH-3: 5′-CGTGTTGAAGGCGAGCA-3′, and GH-4: 5′-GAGAAAGCCTCGCCGTC-3′) were designed for sequence walking. Thus, each clone was divided into six overlapping contigs, which were assembled and extended to one sequence with the SeqMan program, and the 12 clone sequences were aligned and then re-assembled to one or more copies for each accession with the SeqMan and MegAlign program.

### Single nucleotide polymorphisms

SNPs were identified using the DNAStar software.

### Gene mapping

The genotypes of 150 DHLs were determined by PCR–RFLP, and the software Map Manage QTLb20 was used for the gene mapping.

### Population structure and association analysis

Population structure of the historical population was analyzed using Structure 2.3.2 software based on the data of 83 SSR markers (Zhang et al. 2011). Structure produces a Q matrix that lists the estimated membership coefficients for each individual in each cluster. The estimated Q matrices were used in a subsequent association analysis. All polymorphisms (including singletons) were tested, and the *P* value for individual polymorphisms was estimated based on 1,000 permutations of the dataset, both for GLM and logistic regression. Polymorphisms with *P* < 0.05 were considered significantly associated with the traits.

## Results

### Chromosome location of *6*-*SFT*-*A2*

As a young polyploid species, hexaploid wheat has a complex genome. Based on sequence differences in *6*-*SFT* among genomes, A genome-specific primer pair (F2/R2) was designed for chromosome location. The A, B(S), and D genomic donor species of common wheat, and the nulli-tetrasomic lines of Chinese Spring were used to gain insights on the origin and evolution of *6*-*SFT*. PCR results showed that *6*-*SFT*-*A2* was located on chromosomes 4A (Supplementary Fig. S1).

### Nucleotide diversity and haplotypes in the *6*-*SFT*-*A2* region

*6*-*SFT*-*A2* sequences were identified in a small diversity panel consisting of 24 wheat accessions selected from the historical population by molecular markers. The full length of *6*-*SFT*-*A2* is 3,149 bp, including four exons and three introns. Primer F2/R2 covers a 2,663 bp region of *6*-*SFT*-*A2*, including intron 2 (596 bp), exon 3 (858 bp), intron 3 (486 bp), exon 4 (703 bp), and 3′-UTR (20 bp). Eleven SNPs were identified in the whole region of *6*-*SFT*-*A2* with an average of 1 SNP/242 bp (Supplementary Table S2). Two InDel were present. The frequency of SNP in the noncoding region (1 SNP/276 bp) was slightly lower than that in the coding region (1 SNP/223 bp). Among the detected SNPs, eight were transitions and three were transversions. The transition/transversion ratio was 2.7(Supplementary Table S3).

The *6*-*SFT*-*A2* alignment spanning the entire 2,663 bp included 13 sites with alignment gaps (Supplementary Table S3). Four and three SNPs were detected in exons 3 and 4, respectively. Four SNPs were identified in intron 2 and intron 3, and two InDel were in intron 3. Three *6*-*SFT*-*A2* haplotypes, designated *Hap*I, *Hap*II, and *Hap*III, were identified based on the 13 SNP/InDel sites (Supplementary Fig. S2, Table S3).

### Development of CAPS markers for *6*-*SFT*-*A2*

**Fig. 1:**
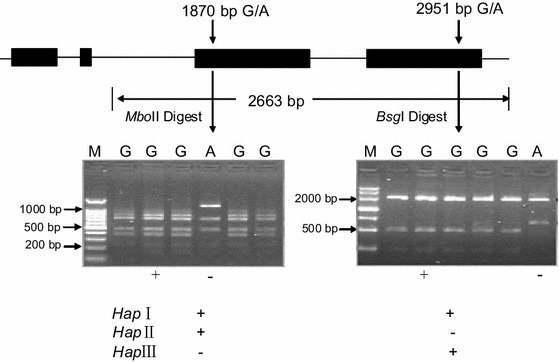
Sketch map of CAPS marker development from *6*-*SFT*-*A2.* Two CAPS markers based on two polymorphisms were used to identify the gene haplotypes. A CAPS marker was developed based on SNP-1870 (G-A). Digestion of the amplified 2,663 bp fragment with *Mbo*II produced fragments of 762 bp/681 bp/465 bp/375 bp/225 bp/155 bp for accessions with SNP-2606G, and 1,137 bp/681 bp/465 bp/225 bp/155 bp with SNP-2606A. M, 100 bp DNA Ladder (TransGen, Beijing, China). The other CAPS marker was developed based on SNP-2951 (G-A). Digestion of the amplified 2,663 bp fragment with *Bsg*I produced fragments of 1,955 bp/510 bp/1,198 bp for accessions with SNP-1955G, and 1,955 bp/708 bp with SNP-1955A. M, Marker III (TransGen, Beijing, China)

Two cleaved amplified polymorphic sequence (CAPS) markers were developed based on the SNPs at 1,870 bp and 2,951 bp to distinguish the three haplotypes (Fig. 1). The nucleotide diversities in *6*-*SFT*-*A2* produced two restriction enzyme sites. One was a *Mbo*II recognition site at SNP-1870-G, but not at SNP-1870-A; the other was a *Bsg*I recognition site at SNP-2951-G, but not at SNP-2951-A (Fig. 1). The two SNPs provided opportunities for developing two CAPS markers to differentiate the *6*-*SFT*-*A2* alleles. In order to discriminate the orthologous genomic sequences, the A genome-specific primer set F2/R2 was used to amplify the 2,663 bp fragment of *6*-*SFT*-*A2* from all accessions; the PCR product was then digested by *Mbo*II, and a length polymorphism, 762 bp/681 bp/465 bp/375 bp/225 bp/155 bp versus 1,137 bp/681 bp/465 bp/225 bp/155 bp, was generated and was easily distinguished on agarose gels (Fig. 1). When the same product was digested by *Bsg*I, the resultant length polymorphism 1,955 bp/510 bp/198 bp versus 1,955 bp/708 bp was again easily distinguished on agarose gels (Fig. 1).

### Association between *6*-*SFT*-*A2* haplotypes and TGW in the historical population

To investigate the effects of the three haplotypes on TGW, 154 Chinese wheat cultivars from the historical population were genotyped using the two CAPS markers for *6*-*SFT*-*A2*. Based on the population structure analysis of Zhang et al. (2011), these accessions were clustered into four subsets by the Structure 2.3.2 software. Thus, we accepted the kinship value as *K* = 4.

Association analyses between *6*-*SFT*-*A2* haplotypes and TGW were performed. The significant associations were only detected from both rainfed trials in 2008 and 2009 (Table 1). The mean TGW of *Hap*III was higher than that of both *Hap*I and *Hap*II. It indicated that *6*-*SFT*-*A2 Hap*III was a superior allele for TGW under rainfed conditions in the historical population.

**Table 1:** Association of *6*-*SFT*-*A2* haplotypes with TGW in the historical population

Year	Haplotype	*N* ^a^	Mean ± SD^b^ (g)	*F* value^c^	*P* value
2008	*Hap*I	39	34.79 ± 4.79	2.85	0.027*
	*Hap*II	38	33.02 ± 5.60		
	*Hap*III	74	35.60 ± 4.87		
2009	*Hap*I	39	37.34 ± 5.66	3.04	0.031*
	*Hap*II	38	36.66 ± 5.36		
	*Hap*III	74	39.79 ± 5.44		

* Significance at *P* ≤ 0.05 level

^a^ *N* number of accession

^b^SD standard deviation

^c^ *F* value based on one-way ANOVA

### Association of *6*-*SFT*-*A2* haplotypes with TGW in a DH population

DNA sequencing showed that a SNP (G/A) at 1,870 bp of *6*-*SFT*-*A2* in Hanxuan 10 distinguished it from Lumai 14 after *Mbo*II digesting. The CAPS marker was used to genotype the DHLs. A total of 83 lines had the haplotype of Hanxuan 10, whereas the others gave the same pattern as Lumai 14.

To assess the effects of the two haplotypes on TGW, we analyzed TGW differences in 150 DHLs grown under rainfed and irrigated conditions over six growing seasons. The results of association analysis showed that TGW was associated with haplotypes, with significant differences between the Hanxuan 10 genotype (*Hap*I) and the Lumai 14 genotype (*Hap*III) in both water regimes except for irrigated conditions in 2001 and 2011 (Table 2). All TGW of lines with *Hap*III were significantly higher than that of lines with *Hap*I.

**Table 2:** Comparison of TGW associated with *6*-*SFT*-*A2* haplotypes in DH population in multi-environments

Year	Haplotype	DS			WW		
		*N*	Mean ± SD (g)	*P* value	*N*	Mean ± SD (g)	*P* value
2001	*Hap*I	83	30.57 ± 6.28	0.001***	83	33.81 ± 5.68	0.064
	*Hap*III	63	33.81 ± 5.10		63	35.70 ± 6.44	
2005	*Hap*I	83	34.02 ± 6.49	0.002**	83	30.70 ± 6.11	0.023*
	*Hap*III	63	37.22 ± 5.67		63	32.98 ± 5.72	
2006	*Hap*I	71	32.40 ± 4.82	0.024*	82	30.24 ± 5.47	0.016*
	*Hap*III	55	34.34 ± 4.56		63	32.95 ± 6.13	
2009	*Hap*I	83	38.06 ± 5.27	0.002**	83	40.66 ± 4.81	0.040*
	*Hap*III	63	40.65 ± 4.34		63	42.25 ± 4.35	
2010	*Hap*I	82	32.89 ± 5.04	0.002**	83	34.32 ± 5.41	0.001***
	*Hap*III	59	35.66 ± 4.52		63	36.91 ± 4.33	
2011	*Hap*I	83	37.82 ± 4.21	0.021*	83	38.07 ± 4.64	0.105
	*Hap*III	63	39.43 ± 4.06		63	39.40 ± 5.14	

*DS* drought stressed, *WW* well-watered, *N* number of DH lines

*, **, *** Significance at *P* ≤ 0.05, *P* ≤ 0.01, and *P* ≤ 0.001, respectively

### Gene mapping

Using the DH population derived from the cross Hanxuan 10 × Lumai 14, linkage analysis showed that *6*-*SFT*-*A2* was mapped on chromosome 4A in a region flanked by markers *P2454.3* (4.5 cM) and *P3465.1* (29.1 cM) (Supplementary Fig. S3). Using the same DH population, QTL for TGW were also identified in the same or adjacent interval flanking by markers *P2454.3* and *P3465.1* on chromosome 4A (Su et al. 2009; Yang et al. 2007).

### Distribution of *6*-*SFT*-*A2* haplotype frequencies in the historical cultivars

**Fig. 2:**
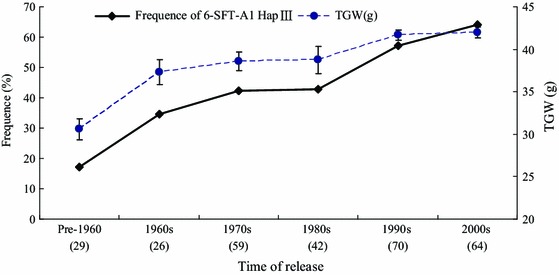
Frequencies of *6*-*SFT*-*A2* *Hap*III in Chinese wheat cultivars released in different decades. *Bars* indicate standard errors

The frequencies of *6*-*SFT*-*A2* *Hap*III in the historical population of 295 accessions were detected in 10-year intervals (pre-1960, 1960s, 1970s, 1980s, 1990s, and 2000s). The released time of the population accessions was more than six decades (Supplementary Table S1). In landraces and cultivars released before 1960 (pre-1960), the frequency of *Hap*III was 23.1 %. Over subsequent periods, the frequency of *Hap*III gradually increased to 64.1 % in cultivars released during the 2000s (Fig. 2). The TGW exhibited increased from pre-1960 to the 2000s. This strongly indicates that *6*-*SFT*-*A2 Hap*III was positively selected in breeding program and it is likely a beneficial haplotype for grain yield improvement in wheat.

### Geographic distribution of cultivars with *6*-*SFT*-*A2* haplotypes in northern and central China

**Fig. 3:**
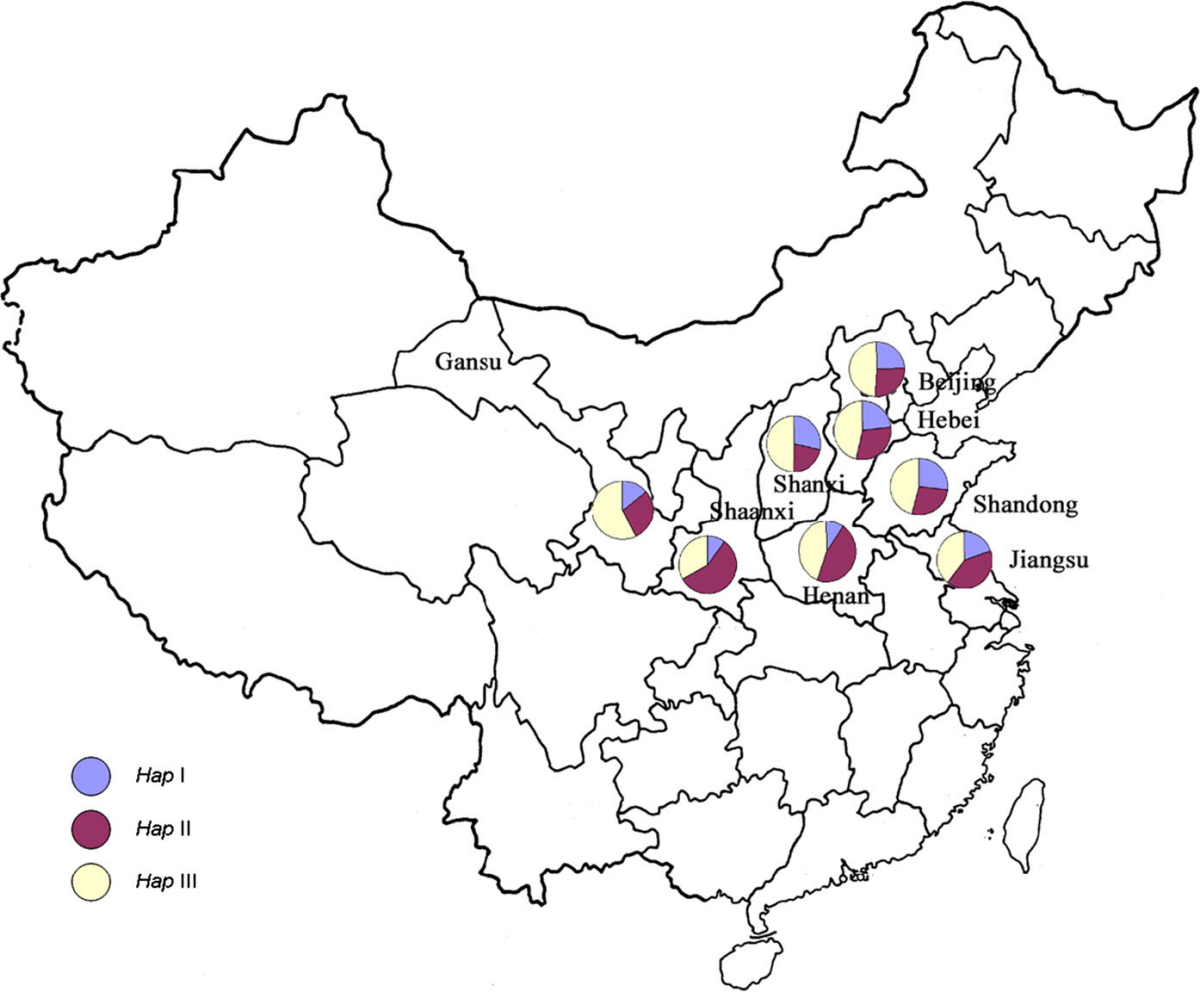
Geographic distribution of cultivars with *6*-*SFT*-*A2* haplotypes in eight provinces in northern and central China

The 272 accessions were collected from eight provinces in northern and central China. The geographic distribution of *6*-*SFT*-*A2* haplotypes is shown in Fig. 3. *Hap*III was the most frequent haplotype in these provinces except for Shaanxi. The *Hap*III frequencies across the eight provinces were 57.1 % (Gansu), 55.8 % (Beijing), 50.0 % (Shanxi), 47.1 % (Hebei), 46.2 % (Shandong), 45.0 % (Henan), 40.0 % (Jiangsu), and 33.3 % (Shaanxi). With increasing latitude, *Hap*II frequencies displayed a decline trend, while *Hap*I frequencies showed a slight increase.

## Discussion

### Identifying causative polymorphisms for TGW in wheat

Sucrose–fructan 6-fructosyltransferase (6-SFT) transfers fructosyl residues to fructan in (2–6) linkages in wheat; these are by far the prevailing linkages in wheat fructans. Therefore, most of the carbon flux from sucrose to fructan in wheat is mediated by 6-SFT (Duchateau et al. 1995; Vijn and Smeekens 1999). Based on the SNPs detected in the *6*-*SFT*-*A2* sequence three haplotypes, *Hap*I, *Hap*II, and *Hap*III, were identified among wheat accessions. Association analysis revealed that *6*-*SFT*-*A2* was associated with TGW both in a historical population and in a DH population. *Hap*III possessed a significantly positive effect on TGW and, therefore, should be a beneficial allele for improving grain yield. Xue et al. (2008) found that the mRNA levels of *6*-*SFT* in wheat stems were positively correlated with stem total WSC and fructan concentrations. Therefore, the differential effects of the three *6*-*SFT*-*A2* haplotypes on grain weight detected in the present study might be caused by different contributions to fructan biosynthesis. Studies of gene expression and enzyme activity of *6*-*SFT*-*A2* would further explore the allelic effects of these polymorphisms.

The *6*-*SFT*-*A2* gene was located on chromosome 4A, between markers *P2454.3* and *P3465.1* in the DH population derived from a cross of Hanxuan 10 × Lumai 14. Using the same DH population, quantitative trait loci (QTL) for TGW were identified in the same or adjacent region on chromosome 4A (Su et al. 2009; Yang et al. 2007). Therefore, we speculate that *6*-*SFT*-*A2* might be the underlying gene for these QTL. *Hap*III appeared to be a superior allele for TGW. Some cultivars with *Hap*II also showed high TGW, suggesting the presence of other genes/QTL which associated with grain weight.

### Verification of association analysis results

A common concern regarding association analysis is false correlation between molecular markers and traits. The population structure of natural populations is that LD can be caused by admixture of subpopulations, which leads to false-positive results if not correctly controlled in statistical analysis (Pritchard 2001; Yu and  Buckler 2006). The complex breeding history of many crops and limited gene flow between subpopulations have created complex stratifications that complicate association studies (Sharbel et al. 2000). To reduce this risk, estimates of population structure must be included in association analysis. However, if the distribution of functional alleles is highly correlated with population structure, statistical control of population structure can result in false-negatives, particularly for small size samples (Yu and Buckler 2006). Hence, to reduce the risks of both false-positives and false-negatives, we used not only a historical cultivar set, but also a DH population in the association analysis to validate the relationships between TGW and a functional marker for *6*-*SFT*-*A2*, and TGW and QTL, identified previously in the same DH population by linkage mapping (Su et al. 2009; Yang et al. 2007).

Compared with the DH population, an effect of *6*-*SFT*-*A2* on TGW could not be detected in the historical cultivar set grown under irrigated conditions. The reason for this could be that the effect of *6*-*SFT*-*A2* was confounded by other genes for TGW, due to the higher overall genetic diversity. The effect of *6*-*SFT*-*A2* was detected under rainfed conditions in both the historical and DH populations. In addition to being carbohydrate reserves, fructans are thought to have roles in protecting plants against environmental stresses such as drought. Previous research also showed that fructan content and its metabolism were related to frost and drought tolerance (Hendry 1993; Pilon-Smits et al. 1995; Yoshida et al. 1998; De Roover et al. 2000; Xue et al. 2008). Thus, association analysis can be more reliable for evaluating the relationships between important agronomic traits and markers when using cultivar sets and genetic populations, such as DH population.

### Developing functional markers based on gene sequence polymorphism

Functional or perfect markers derived from polymorphic sites within genes conferring specific phenotypes (Andersen and Lübberstedt 2003; Bagge et al. 2007) are ideal for marker-assisted breeding. For example, genes for starch biosynthesis, such as ADP-glucose phosphorylase (*Agp*-*L*), sucrose transporter (*SUT*), and starch synthase I (*SSI*), were correlated with changes in grain yield (Blake et al. 2004). Conversion of SNPs to CAPS markers facilitates their application in plant genetics and breeding. CAPS markers can be assayed by a simple process: PCR, restriction enzyme digestion, and agarose gel electrophoresis. TGW is an agronomically important trait that continuously attracts the attention of breeders as a generally accepted component of yield. Genes contributing to high TGW could be targets for molecular selection during wheat improvement. Therefore, we designed a genome-specific primer set to differentiate the orthologous sequences, and then, based on the sequence differences between the haplotypes of *6*-*SFT*-*A2* in different varieties, two CAPS markers were developed. Their potential value for selection of TGW was validated by association analysis. Two CAPS markers are codominant and allow rapid assays of large numbers of samples in a simple, rapid, and low-cost procedure, which is available in most molecular biology and/or plant breeding laboratories.

## Electronic supplementary material

Below is the link to the electronic supplementary material.

Supplementary material 1 (XLS 50 kb)

Supplementary material 2 (DOC 81 kb)

Supplementary material 3 (DOC 114 kb)

## Abbreviations

CAPS: Cleaved amplified polymorphic sequence
DH: Doubled haploid
DHLs: Doubled haploid lines
DS: Drought stress
InDel: Insertion/deletion
QTL: Quantitative trait loci
SNP: Single nucleotide polymorphism
TGW: Thousand-grain weight
WSC: Water-soluble carbohydrates
WW: Well-watered
