# Supplementary material for: Identification and development of a functional marker from 6-SFT-A2 associated with grain weight in wheat
Source: Mol Breed. 2015 Jan 30;35(2):63. doi: 10.1007/s11032-015-0266-9 (PMC4311048; doi:10.1007/s11032-015-0266-9)
Supplement: Supplementary file 2 — Supplementary material 2 (DOC 81 kb) [file 11032_2015_266_MOESM2_ESM.doc]

Table S2 Nucleotide acid variation in wheat *6-SFT-A2* gene

| Region | Length  (bp) | SNP | | InDel | |
| --- | --- | --- | --- | --- | --- |
|  |  | Number | Frequency | Number | Frequency |
| Noncoding | 1102 | 4 | 1/276 | 2 | 1/551 |
| Coding | 1561 | 7 | 1/223 | 0 | 0 |
| All | 2663 | 11 | 1/242 | 2 | 1/1332 |

Table S3 Haplotypes of *6-SFT*-*A2* based on single nucleotide polymorphisms in genomic

sequences

| No. | Site(bp)^a^ | Region | Type | Single nucleotide mutation | *Hap*I | *Hap*II | *Hap*III |
| --- | --- | --- | --- | --- | --- | --- | --- |
| 1 | 600 | Intron 2 | SNP | G/A | G | G | A |
| 2 | 730 | Intron 2 | SNP | T/C | T | C | T |
| 3 | 807 | Intron 2 | SNP | T/A | C | A | C |
| 4 | 858 | Intron 2 | SNP | C/A | C | C | A |
| 5 | 1207 | Exon 3 | SNP | G/A | G | A | A |
| 6 | 1237 | Exon 3 | SNP | A/T | A | C | T |
| 7 | 1591 | Exon 3 | SNP | C/T | C | C | T |
| 8 | 1870 | Exon 3 | SNP | G/A | G | G | A |
| 9 | 2053 | Intron 3 | InDel | T/0 | T | 0 | T |
| 10 | 2056 | Intron 3 | InDel | 0/C | 0 | C | 0 |
| 11 | 2546 | Exon 4 | SNP | C/T | C | C | T |
| 12 | 2918 | Exon 4 | SNP | G/C | G | G | C |
| 13 | 2951 | Exon 4 | SNP | G/A | G | A | G |

^a^ With reference to ATG translation start codon
