# Supplementary material for: Identification and development of a functional marker from 6-SFT-A2 associated with grain weight in wheat
Source: Mol Breed. 2015 Jan 30;35(2):63. doi: 10.1007/s11032-015-0266-9 (PMC4311048; doi:10.1007/s11032-015-0266-9)
Supplement: Supplementary file 3 — Supplementary material 3 (DOC 114 kb) [file 11032_2015_266_MOESM3_ESM.doc]

**Fig. S1 PCR-based chromosome location of *6-SFT*-*A2* using genome-specific primer pair F2/R2*.*** Primers specific for *6-SFT*-*A2* successfully amplified the expected 2,663 bp fragment from *T. urartu* (AA), *T. dicoccoides* (AABB), *T. durum* (AABB)，Hanxuan 10, Lumai 14, Opata M85, W7984, Chinese Spring , N4BT4A , N4DT4B, but not from *Ae. speltoides* (SS, closely related to the B genome), *Ae. tauschii* (DD), N4AT4B and N4AT4D, indicating that *6-SFT*-*A2* is located on chromosome 4A. M, Marker III (TransGen, Beijing, China)

**Fig. S2 Phylogenetic tree of 24 wheat accessions based on 13 SNP/InDel in *6-SFT-A2* gene.** Three *6-SFT*-*A2* haplotypes, designated as *Hap*I, *Hap*II and *Hap*III, were identified

**Fig. S3 Linkage mapping of *6-SFT-A2* on wheat chromosome 4A based on a doubled haploid (DH) population derived from a cross of Hanxuan 10 × Lumai 14.** The *6-SFT-A2* marker was located on chromosome 4A and flanked by SSR markers *P2454.3* and *P3465.1*
